# Calling Names

**DOI:** 10.1523/ENEURO.0314-20.2020

**Published:** 2020-08-07

**Authors:** Christophe Bernard

I am delighted to see two excellent commentaries published in *eNeuro*. György Buzsáki contacted me because, 20 years ago, he had tried to publish a commentary regarding the way behavioral-cognitive neuroscience is conducted; it was rejected. Twenty years later, György sent the same commentary verbatim to the same journal, because he considered the ideas still topical. After review, György decided not to perform the requested revisions because he thought that the original text “was the product of the time.” He submitted it to *eNeuro*, and we decided to publish it as is (Buzsáki, 2020) because I thought that his arguments are very interesting and important to discuss. György argues that behavioral-cognitive neuroscience should invent its own vocabulary and be based on a mechanistic approach. I shall not express an opinion, just that I consider this idea worthy of debate. His arguments are as important to discuss now as they were 20 years ago.

However, since it can be seen as a strong attack on the way behavioral-cognitive neuroscience is done, I have asked David Poeppel (Poeppel and Adolfi, 2020), a leader in cognitive neuroscience, to react to György’s ideas. Both commentaries contain fantastic material and should be read in sequence, starting with György’s. As you will see, György and David don’t call each other names (they are friends). I hope you will agree that this is a very interesting debate and worth developing further (you can contact me if you have some suggestions).

A core issue that emerges from both texts is that of “naming.” Both texts acknowledge the importance of the “names” we give to describe observables in science. I would like to add my two cents to the debate and go a bit further regarding the issue of naming. Already as a kid, I started to question everything I was told and taught. I vividly remember when I was 12 or so, panicking at the idea that I would lose words from my vocabulary. I would see something red, but I would not be able to remember the word “red” anymore, and thus I would not be able to communicate with others. This fear led me to question the nature of language and the meaning of words. Who decided that this color would be named red, and why? What if the meaning a word carries for me has a different meaning for someone else? How can we communicate in these conditions? At 12, my questioning was limited to the possibility that a friend would call something blue, while I would call it red, as well as the possibility of permanently losing some words from my vocabulary. Fortunately, I finally realized the societal and communal nature of the meaning of words. Now, I am back to square one with words and naming in science.

Experimental science is nothing but trying to make sense of observations. Since we need to describe what we observe, we need to name what we see. As Richard III said: “I cry thee mercy, then, for I did think That thou hadst called me all these bitter names.” Naming in science has a long tradition, and many of us like to invent or use catchy names. This is a field in itself. Since defining *X-omics* is highly fashionable, I do not want to deviate from the rule and propose a new field: *nomenomics*, and since proposing a *X-pathy* is as fashionable and catchy, let us also have the *nomenopathy* (obviously, I do suffer from it). Often, naming is anything but innocuous. Using a word from the common vocabulary will necessarily carry a value with it. I remember laughing out loud (literally) when I heard many years ago a neuroscientist talking about the *cornichon* subunit of AMPA receptors. In French, it means both pickle and, when you talk about someone, fool. Naming it *cornichon* had a strong impact on me. Although I would be hard-pressed to recall other subunits of AMPA receptors, I still do remember the “fool” subunit. Naming it *cornichon* did facilitate memorizing it, and perhaps did not lead the field astray, i.e., it was innocuous. In contrast, this is not the case for “imbalance between excitation and inhibition” in my field, epilepsy. The three nouns possess a very strong valence. After all, you must maintain balance in all things, refrain from too much excitation, and above all not becoming disinhibited. These three names are highly emotionally charged, and I could write a book on how it influenced epilepsy research and treatments. If red has been defined as having a 625- to 740-nm wavelength, agreed by all, what is the definition of a balance (what is the metric?), of excitation, of inhibition in the context of neuroscience? The concepts are catchy, and I have been using them extensively in my scientific publications. Epilepsy researchers and clinicians will nod knowingly if you speak of imbalance between excitation and inhibition, as if we all know what we are talking about, and thus have a common language. Of course, the fact that these nouns refer to concepts rather than to a concrete object does not help. There is no single, generally agreed metric for excitation, which we can measure and report. Derivatives, such as hypoexcitability, overexcitability, or hyperexcitability appeared, which have no precise definition either. The use of non-concrete terms may constitute a significant impediment to the progress of science. A cornichon is a tangible subunit, but we may all be *cornichons* to take for granted non-concrete and high-valence words. I argue that it biases our reasoning (words with high valence shape our thoughts whether we wish or not). Perhaps, as György wrote, we should invent a vocabulary for observations that rely on interpretations (e.g., greediness in behavioral neuroscience). The major hurdle is to dissociate something that is tangible from the non-tangible.

Naming can have important effects. Neurons firing at a higher rate in a specific portion of the environment are called place cells. Notwithstanding the strong valence that “place” has for us humans, it infers that some cells code for space in the brain, and that the brain represents space. However, we should remember that this remains a hypothesis on which one can build a theory of brain function (there is nothing wrong with this). There is no doubt that some cells fire that way in a specific location of the environment, this is concrete. Do they code a place, which assumes that the brain builds a map of the space? This remains to be demonstrated. To paraphrase György, what is the null hypothesis of a place cell? Here lies another danger in science: a hypothesis is often taken as ground truth, thus imposing strong constraints on the way we do science. After place cells, papers reported the existence of grid cells, time cells, border cells, and even Halle Berry and Simpsons neurons in the human brain. Catchy names. Did they fuel the field, generating hypotheses and theories? Definitely, and significant advances were made. Do they constrain us in our capacity to accurately interpret the observations? Possibly.

Inventing a vocabulary would help, but we will always face the problem of what is tangible and what is not. György being a friend, I can turn his arguments against himself. He argues that “behavioral-cognitive neuroscience … should … define descriptors” (Buzsáki, 2020). But he consistently writes about place cells and field oscillations. Field oscillations are also present in most of my papers. However, if we take a close look, the variations we see in electrophysiological recordings are not oscillations stricto sensu. An oscillation requires the existence of a central value, which is difficult to find when you analyze raw, non-filtered, signals. Has anyone checked the null hypothesis regarding oscillations? Worse, the local electromagnetic field generated by the movement of charges in the brain cannot be exactly measured because a true reference cannot be obtained. The field we measure is, at best, a poor approximation of the real field. Yet, naming these events “oscillations” has a strong impact, as we can relate them to mathematical properties (such as phase, cross-frequency coupling, etc.), which are grounded in reality. György, what if “oscillation” is an inaccurate descriptor of the reality? Does it constrain our imagination and prevent us to better understand the brain?

Here I am, back to a 12-year-old again (or still there, as my wife and kids would say), questioning the naming of things. As you can see, these two commentaries got me excited, I lost my inhibition, and provided an imbalanced view, or not. You can call me names now.
